# A comparison between p16-positive head and neck cancer of unknown primary (HPV-HNCUP) and oropharyngeal squamous cell carcinoma (HPV-OPSCC): are they the same disease?

**DOI:** 10.1007/s00405-023-08115-5

**Published:** 2023-07-28

**Authors:** Matthias Balk, Robin Rupp, Matti Sievert, Konstantinos Mantsopoulos, Moritz Allner, Philipp Grundtner, Sarina K. Mueller, Markus Eckstein, Heinrich Iro, Markus Hecht, Antoniu-Oreste Gostian

**Affiliations:** 1 Comprehensive Cancer Center Erlangen-Europäische Metropolregion Nürnberg, Department of Otolaryngology, Head & Neck Surgery, Friedrich-Alexander University Erlangen-Nürnberg (FAU), University Hospital Erlangen, Waldstraße 1, 91054 Erlangen, Germany; 2 Comprehensive Cancer Center Erlangen-Europäische Metropolregion Nürnberg, Department of Pathology, Friedrich-Alexander University Erlangen-Nürnberg (FAU), University Hospital Erlangen, Krankenhausstraße 8-10, 91054 Erlangen, Germany; 3 Comprehensive Cancer Center Erlangen-Europäische Metropolregion Nürnberg, Department of Radiation Oncology, Friedrich-Alexander University Erlangen-Nürnberg (FAU), University Hospital Erlangen, Universitätsstraße 27, 91054 Erlangen, Germany

**Keywords:** Cancer of unknown primary, Head and neck cancer, Neck dissection, Oropharyngeal cancer, HPV-HNCUP, HPV-OPSCC

## Abstract

**Introduction:**

The following study aimed to answer the question if HPV-HNCUP and HPV-OPSCC are the same disease. Propensity score matching (PSM) was used to compare the oncological outcomes of both groups, in particular the 5-year overall survival rate (OS), the 5-year disease specific survival rate (DSS) and the 5-year progression free survival rate (PFS).

**Materials and methods:**

Firstly, between January 1st, 2007, and March 31st, 2020 a total of 131 patients were treated with HNCUP at our Department. Out of these, 21 patients with a confirmed positive p16 status were referred to surgery followed by adjuvant therapy. Secondly, between January 1st, 2000, and January 31st, 2017, a total of 1596 patients were treated with an OPSSC at our Department. Out of these, 126 patients with a confirmed positive p16 status were referred to surgery followed by adjuvant therapy. After PSM, 84 patients with HPV-OPSCC and 21 HPV-HNCUP remained in the study for further comparison.

**Results:**

The OS was 63.5% (95% CI 39.4–87.6) for HPV-HNCUP and 88.9% (95% CI 90.4–100.0) for HPV-OPSCC patients and therefore, significantly lower for the first mentioned (*p* = 0.013). The DSS was also significantly impaired for HPV-HNCUP (71.0%, 95% CI 46.3–95.7), in comparison with HPV-OPSCC patients (95.5%, 95% CI 90.4–100.0; *p* = 0.002). The PFS for HPV-HNCUP patients was lower (75.6%, 95% CI 54.0–97.2) yet not significantly different to HPV-OPSCC (90.4%, 95% CI 83.5–97.3; *p* = 0.067).

**Conclusions:**

The results presented demonstrate a significant reduced OS and DSS for HPV-HNCUP patients. Accordingly, in our study HPV-HNCUP and HPV-OPSCC are two different entities with a different oncological outcome.

**Supplementary Information:**

The online version contains supplementary material available at 10.1007/s00405-023-08115-5.

## Introduction

We see an increasing incidence for oropharyngeal carcinoma in the last decades, a fact that is due to the increasing number of detected infections with human papilloma virus (HPV) [1, 2]. In 2018, 100,500 new cases were registered worldwide [3], of which in western countries the prevalence for HPV-associated oropharyngeal carcinoma (HPV-OPSCC) ranges between 35 and 80% [4, 5]. This wide range is also reflected in the prevalence of HPV-associated cancer of unknown primary (HPV-HNCUP), which varies in the literature between 22 and 91% [6–10]. HNCUP is a disease in which metastasis to the neck occurs and the primary tumor is not detected after a thorough clinical examination and radiological imaging.

The distinction between HPV-associated and non-associated carcinomas is necessary because on the one hand the clinical aspects of the patients change, they are usually younger and do not show the noxious history typical for HPV-negative carcinomas [11], but it is also reflected in a better oncological outcome [12].

The question now arises, especially since Winter et al. and Golusinski et al. were able to show that HPV-HNCUP is often an occult HPV-OPSCC [13, 14], whether both distinct diseases may in fact be the same disease as hypothesized by Schroeder et al. [15]. Since many studies on HNCUP involve inhomogeneous patient populations with different therapies at different institutions, our intention was to investigate this hypothesis in the following study using a collective treated at one center with comparable therapies in each case. As primary objective served the oncologic outcome, in particular the 5-year overall survival rate. The recurrence of disease, i.e., local and/or regional metastases and distant metastases, the 5-year disease specific survival rate and the 5-year progression free survival rate were defined as secondary objectives.

## Patients and methods

We conducted this retrospective cohort study at a single tertiary referral and academic cancer center. It was approved by the local Ethics Committee (approval number 428_20 Bc) and was carried out according to the Declaration of Helsinki.

All patients diagnosed with a p16 positive CUP-syndrome between January 1st, 2007, and March 31st, 2018, as well as all patients diagnosed with HPV-OPSCC between January 1st, 2000, and January 31st, 2017, were included. The following inclusion criteria were applied: surgical treatment at our institution, histologically confirmed squamous cell carcinoma, confirmed positive p16-status and complete medical and surgical record available. The association with HPV was confirmed by overexpression of the surrogate marker p16INK4a.

The following exclusion criteria were applied: detection of the primary cancer during regular diagnostic work-up, histological types other than squamous cell carcinoma, negative or unknown p16-status or discontinued treatment as well as previous head and neck radiation.

Smoking was defined as current smokers with a smoking history of at least more than 10 pack years, Alcohol consumption was defined as reported daily alcohol intake (yes/no).

The treatment process for CUPs involved first of all a “no-touch”-panendoscopy followed either preferably by a core needle biopsy or in case this is not possible, a node picking of the suspicious lymph node. Afterwards, a positron emission tomography (PET) was performed. If there was still no evidence of the primary cancer, the PET was followed by a CUP-panendoscopy that included a curettage of the nasopharynx, bilateral tonsillectomy and pathological evaluation of the tongue base. Simultaneously, the respective neck dissection was performed on the side of the previously diagnosed malignant lymph node. In case of contralateral suspicious lymph nodes a bilateral neck dissection was performed.

The treatment process of HPV-OPSCC involved primary ablative surgery, either through a transoral, transcervical or combined approach with or without simultaneous microvascular reconstruction and neck dissection of the appropriate nodal basis.

Adjuvant treatment consisted of either radiation therapy or chemoradiation. Indications for chemoradiation were extranodal extension and more than one affected lymph node for HNCUP patients [16]. HPV-OPSCC patients received radiotherapy for the following criteria: ≥ T3 disease, close resection margin (< 5 mm), lymph node metastases without extranodal extension, lymphovascular invasion, or perineural invasion. Concomitant chemotherapy was performed in patients with T4 disease, positive margins, ≥ 3 lymph node metastases, and the presence of extranodal extension.

The classification of the neck dissection was according to Robbins et al. where selective neck dissection denotes preservation of one or more groups of lymph nodes, modified radical neck dissection denotes preservation of one or more non-lymphatic structures and radical neck dissection denotes removal of the sternocleidomastoid muscle, the spinal accessory nerve, and the internal jugular vein besides the lymph node groups [17]. Moreover the Lymph Node Ratio (LNR) was determined and was defined as the number of positive lymph nodes divided by the total number of lymph nodes removed [18].

Radiation techniques included 3D conformal radiation therapy, intensity-modulated radiation therapy (IMRT), or volumetric modulated arc therapy (VMAT). The dosage of radiation was 64 Gray (Gy) in the area of the affected lymph node and 56 Gy in the ipsilateral neck and possible primary sites (i.e. tonsils bilaterally, tongue base and hypopharynx) for HNCUP patients. The contralateral neck received 50 Gy. A dose of 70 Gy was delivered to the nasopharynx. HPV-OPSCC patients received 61 Gy in the primary tumor region and 52.3 Gy in the corresponding lymph node levels. Treatment was delivered either in single doses of 2.0 Gy sequentially in shrinking field technique or as simultaneous integrated boost using single doses of up to 2.3 Gy up to biologically equivalent cumulative doses. The standard approach for concomitant chemotherapy was two cycles of 5-Fluorouracil (800 mg/m^2^ body surface area (BSA) continuous infusion d1-5) in combination with cisplatin (100 mg/m^2^ BSA) or carboplatin (AUC 5) splitted to 3–5 days.

### Outcome measures

Recurrence of disease was defined as local and/or regional tumor recurrence or distant metastasis. As primary objective served the 5-year overall survival rate and was calculated from the date of the neck dissection to the date of death from any cause. As secondary objectives served the 5-year disease specific survival rate which calculated from the date of the neck dissection to the date of death from the disease. Furthermore, the 5-year progression free survival rate which calculated from the date of the neck dissection to the date of progression of the disease. Patients that were still alive at the time of the follow-up cut-off were censored. Follow-up for all patients consisted of a clinical examination with a thorough ultrasound performed by an ENT specialist covering both sides of the neck every 6 weeks in the first year after the disease, every three months in the second and third year and every six months in the fourth and fifth year after finishing the treatment of the disease. A computed tomography scan of the neck and thorax was performed once a year. The tumor stage was determined according to the 8th version of the UICC [19].

### Statistical analysis

Continuous variables were tested for normal distribution using the Kolmogorov–Smirnov test and each variable’s histogram. Consequently, normally distributed parameters are presented as mean ± 1 standard deviation (SD) and compared via independent *T* Tests. In case of non-normally distributed variables, the median [1.; 3. Quartile] is additionally presented, and group comparisons were performed with the Mann–Whitney-*U*-test.

Categorical variables are presented as absolute and relative values (N / %) and compared with the Chi-Square-Test or with the Exact Fischer-Test, whatever applicable. Survival rates were created by using the Kaplan–Meier-Method and compared by the Log-Rank-Test. Overall-, disease-specific and progression free survival values are presented as Kaplan–Meier estimates and 95% confidential intervals (CI).

A Propensity Score Matching was performed to equalize both study groups regarding clinical variables that had the potential to have a confounding influence on the primary and secondary endpoints. The matching was based on the variables: gender, age, nodal stage, adjuvant treatment modality and the use of noxious agents. The PMS was conducted in R using the package and MatchIt [20]. Specifically, we used a cardinality matching approach with a ratio of 1:4 (to retain a substantial proportion of the control group) and a tolerance of 0.125.

Effect sizes for chi^2^-tests are reported as phi in case of nominal variables with 2 levels and Cramer’s *V* for higher order variables. For independent *t* Tests and the Mann–Whitney-*U*-test r was reported as an effect size. A phi/Cramer’s *V*/*r* of 0.1 displays a small effect, 0.3 a medium, and 0.5 a strong effect [21].

A *p* value of less than 0.05 was considered statistically significant. Statistical analysis except of the PSM was performed using IBM SPSS Statistics, version 26.0 (IBM Corp., Armonk, NY, USA).

## Results

### Patient characteristics

Between January 1st, 2007, and March 31st, 2020 a total of 131 patients were treated with a CUP-syndrome at our Department. Out of these, 21 patients with a confirmed positive p16 status were referred to a so-called CUP-panendoscopy with simultaneous neck dissection followed by adjuvant therapy. The entire patient cohort averaged 59.48 years (years) (SD = 9.90 years) and included 17 male patients (81%).

Between January 1st, 2000, and January 31st, 2017, a total of 1596 patients were treated with an OPSSC at out Department. Out of these, 126 patients with a confirmed positive p16 status were referred to a primary ablative surgery with simultaneous neck dissection followed by adjuvant therapy. The entire patient cohort averaged 59.98 years (years) (SD = 9.33 years) and included 91 male patients (72.2%).

Due to differences between groups at baseline, a 1:4 propensity score matching was performed. This resulted for the HPV-OPSCC group in 84 patients, that averaged 58.54 years (years) (SD = 8.66 years) and included 64 male patients (76.2%). The matching variables and the corresponding group comparisons before and after PSM are depicted in Table S1 off the supplementary material.

After PSM, both groups did not differ significantly with respect to gender (*p* = 0.642, phi = 0.045), age (*p* = 0.693, *d* = 0.105), nodal stage (*p* = 0.739, Cramer’s *V* = 0.076), noxious agents (smoking *p* = 0.612, phi = 0.049, alcohol *p* = 0.051, phi = 0.192), the ASA-Score (*p* = 0.240, Cramer’s *V* = 0.165), UICC stage (*p* = 0.340, Cramer’s *V* = 0.143) and extranodal extension (*p *= 0.242, phi = 0.114).

Patients’ characteristics are presented in Table 1 revealing no significant differences between both groups.

**Table 1:** Patients´ characteristics

	CUP (*n* = 21)	Oropharynx (*n* = 84)	Statistical comparison
Gender (*n*, %)			
Male	17 (81.0%)	64 (76.2%)	*p* = 0.642, phi = 0.045
Female	4 (19.0%)	20 (23.8%)	
Age (mean years ± SD)	59.48 ± 9.90	58.54 ± 8.66	*p* = 0.693, *d* = 0.105
Nodal stage (*n*, %)			
pN0	0 (0%)	2 (2.4%)	*p* = 0.739, Cramer’s *V* = 0.076
pN1	18 (87.7%)	68 (81.0%)	
pN2	3 (14.3%)	14 (16.7%)	
No ND	0 (0%)	0 (0%)	
UICC stage (*n*, %)			
I	18 (85.7%)	60 (71.4%)	*p* = 0.340, Cramer’s *V* = 0.143
II	3 (14.3%)	20 (23.8%)	
III	0 (0%)	4 (4.8%)	
Extranodal extension (*n*, %)			
Yes	13 (61.9%)	40 (47.6%)	*p* = 0.242, phi = 0.114
No	8 (38.1%)	44 (52.4%)	
Noxious agents			
Smoking	18 (85.7%)	68 (81.0%)	*p* = 0.612, phi = 0.049
Alcohol	19 (90.5%)	57 (69.5%)	*p* = 0.051, phi = 0.192
ASA-Score			
1	1 (4.8%)	12 (14.3%)	*p* = 0.240, Cramer’s *V* = 0.165
2	14 (66.7%)	59 (70.2%)	
3	6 (28.6%)	13 (15.5%)	

*SD* standard deviation, *UICC* International Union Against Cancer, *ASA* American Society of Anesthesiologists

### Treatment characteristics

All patients were treated with curative neck dissection (selective, modified-radical and radical neck dissection) and adjuvant chemoradiation was performed in 17 patients (81%) whereas 4 patients (19%) underwent only radiotherapy in the HPV-HNCUP cohort. 64 patients (76.2%) of the HPV-OPSCC group received adjuvant chemoradiation and 20 patients (23.8%) received only adjuvant radiation. The median duration from curative tumor removal to the beginning of adjuvant therapy was 42 d [34.50; 51.00] for HPV-HNCUP patients and 47 d [39.00; 55.75] for HPV-OPSCC patients. There was no significant difference between the two groups in any of the treatment characteristics described above (see Table 2).

**Table 2:** Treatment modalities of all patients

	CUP (*n* = 21)	Oropharynx (*n* = 84)	Statistical comparison
Time span			
Neck dissection—adjuvant therapy (median [25.;75.perc])	42.00 [34.50; 51.00]	47.00 [39.0; 55.75]	*p* = 0.468, *r* = 0.071
Surgical treatment modality (*n*, %)			
Selective neck dissection	10 (47.6%)	57 (67.9%)	*p* = 0.139, *V* = 0.194
Modified radical neck dissection	4 (19.0%)	14 (16.7%)	
Radical neck dissection	7 (33.3%)	13 (15.5%)	
Adjuvant treatment modality (*n*, %)			Fisher’s *Z*: *p* = 0.776, phi = 0.045
Radiation therapy	4 (19.0%)	20 (23.8%)	
Chemoradiation therapy	17 (81.0%)	64 (76.2%)	
Radiation dose in Gy (median [25.;75.perc])	**72.00 [70.0; 72.0]**	**64.0 [63.0; 64.0]**	*p* < 0.001*, *r* = 0.508
Chemotherapy (*n*, %)			
Cisplatin/ carboplatin + 5-FU	17 (100.0%)	62 (96.9%)	Fisher’s *Z*: *p* > 0.999, phi = 0.082
Other	0 (0.0%)	2 (3.1%)	
Number of removed lymph nodes (median [25.;75.perc])	**16.00 [11.5; 21.5]**	**31.0 [21.25; 44.50]**	*p* < 0.001*, *r* = 0.434
Lymph node ratio (LNR; median [25.;75.perc])	0.10 [0.070; 0.18]	0.07 [0.04; 0.18]	*p* = 0.022, *r* = 0.223

Bold: A *p* value of less than 0.05 was considered statistically significant

*SD* standard deviation, *Gy* Gray, *5-FU* 5-Fluorouracil, *V* Cramer’s V

*Significant after Bonferroni correction for multiple comparison (*p*_crit_ = 0.007)

In contrast to this, patients with HPV-HNCUP received significantly higher radiation doses compared to patients with HPV-OPSCC (72, 95 Gy [70.0; 72.0] vs. 64, 95 Gy [63.0; 64.0], *p* < 0.001, *r* = 0.508). Furthermore, less lymph nodes were removed from HPV-HNCUP patients (16.00 [11.5; 21.5] vs. 31.00 [21.25; 44.50], *p* < 0.001, *r* = 0.434). This is due to the fact, that HPV-OPSCC patients received the neck dissection bilateral and every HPV-HNCUP patient only on the affected side.

### Oncologic outcomes

**Fig. 1:**
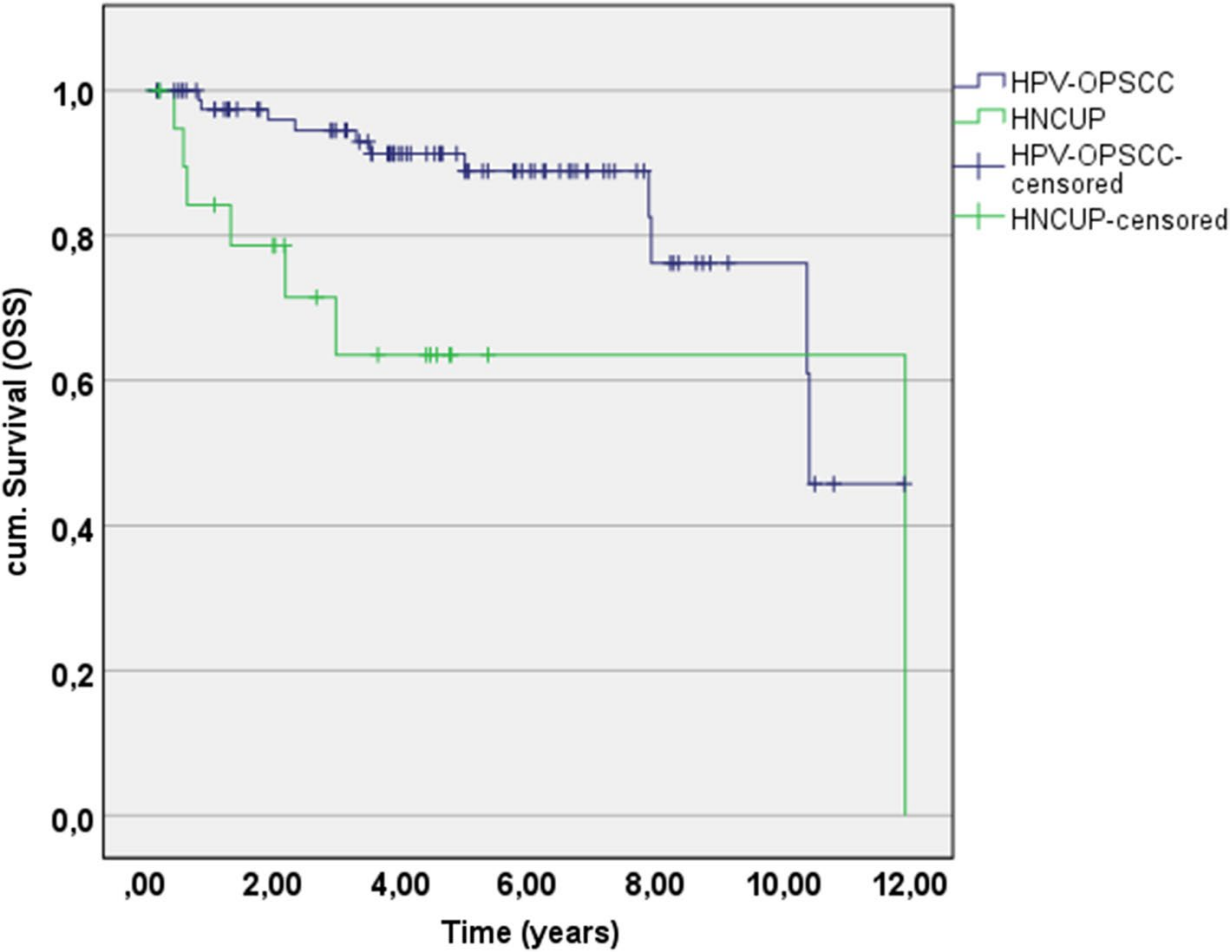
5-year OS

Regarding to the HPV-HNCUP and HPV-OPSCC group, the 5-year overall survival rate was 63.5% (95% CI 39.4–87.6) and 88.9% (95% CI 90.4–100.0; Table 3, Fig. 1) and therefore the OS was significant lower for patients with HPV-HNCUP (*p* = 0.013).

**Table 3:** Oncologic outcome

	CUP (*n* = 21)	Oropharynx (*n* = 84)	Statistical comparison
Recurrence of disease:			
Local and regional metastases	1 (4.8%)	4 (4.8%)	Fisher’s *z*: *p* > 0.999, phi < 0.001
Distant metastases	2 (9.5%)	6 (7.1%)	Fisher’s *z*: *p* = 0.659, phi = 0.036
5-year OS rate			
Events (death) 5 Jahre	6 (23.8%)	7 (8.3%)	
KM estimate (95% CI)	**63.5% [39.4; 87.6]**	**88.9% [80.9; 96.9]**	Log Rank: *p* = 0.013*
5-year DSS rate			
Events (death) 5 year	4 (19.0%)	3 (3.6%)	
KM estimate (95% CI)	**71.0% [46.3; 95.7]**	**95.5% [90.4; 100.0]**	Log Rank: *p* = 0.002*
5-year PFS rate			
Events (death) 5 year	4 (19%)	7 (8.3%)	
KM estimate (95% CI)	75.6% [54.0; 97.2]	90.4% [83.5; 97.3]	Log Rank: *p* = 0.067

Bold: A *p* value of less than 0.05 was considered statistically significant

*OS* overall survival, *DSS* disease-specific survival, *PFS* progression-free survival, *KM* Kaplan–Meier

*Significant after Bonferroni correction for multiple comparison (*p*_crit_ = 0.017)

**Fig. 2:**
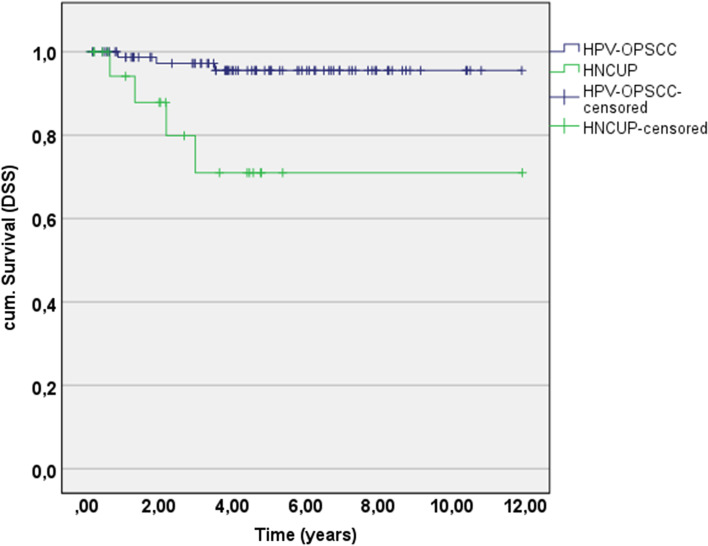
5-year DSS

The 5-year disease-specific survival rate was also significantly lowered for HPV-HNCUP patients (71.0%, 95% CI 46.3–95.7), in comparison with HPV-OPSCC patients (95.5%, 95% CI 90.4–100.0; *p* = 0.002; Table 3, Fig. 2).

**Fig. 3:**
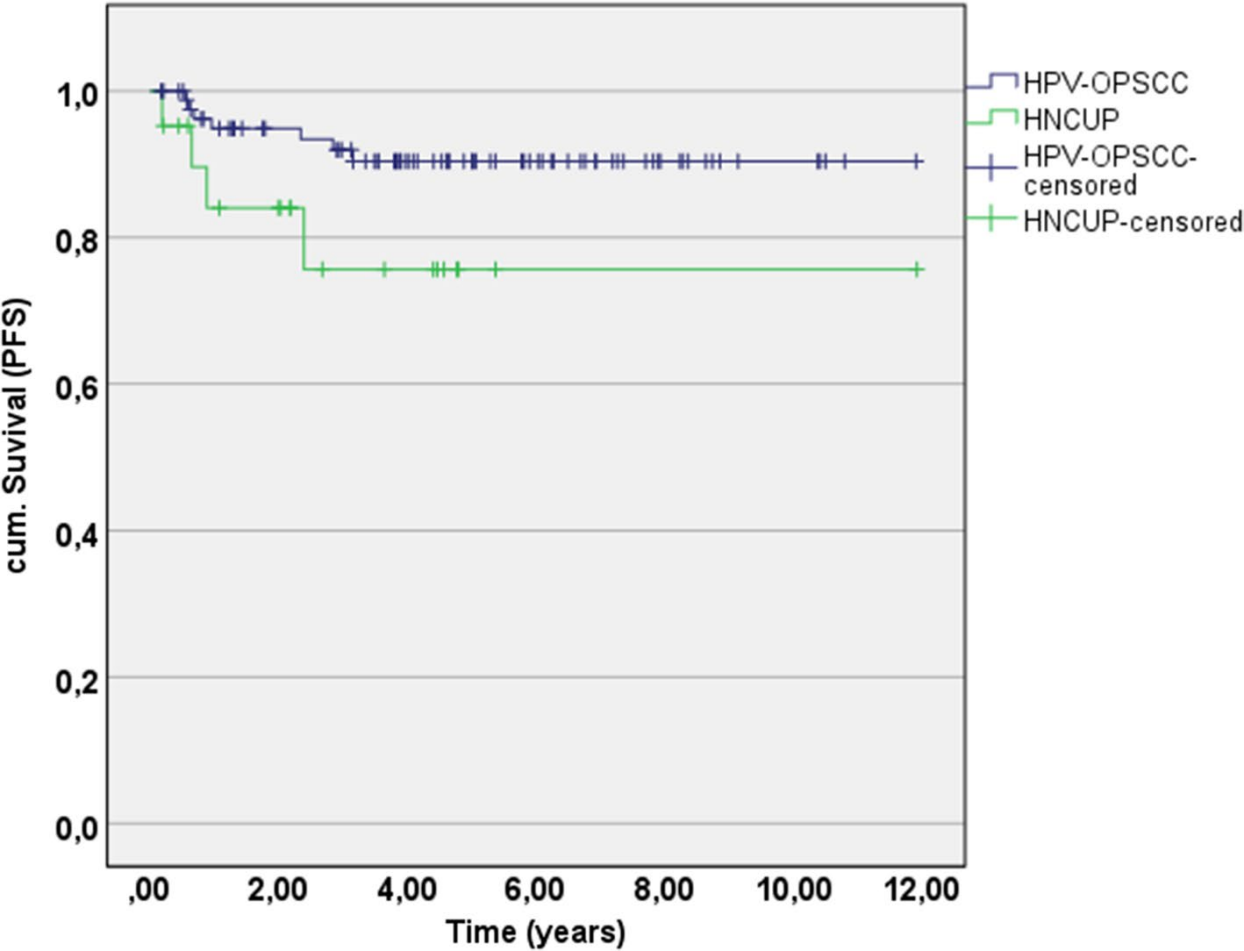
5-year PFS

Finally, the 5-year progression-free survival rate for HPV-HNCUP patients was lower (75.6%, 95% CI 54.0–97.2) yet not significantly different compared to the HPV-OPSCC group (90.4%, 95% CI 83.5–97.3; *p* = 0.067; Table 3, Fig. 3).

## Discussion

The previously reported results with a 5-year OS of 63.5%, a 5-year DSS of 71% and a 5-year PFS of 75.6% for the HPV-HNCUP patients are in terms for the OS worse in comparison to reported outcomes that range from 82.2 to 92% [8, 22–24]. The HPV-OPSCC patients showed significantly better oncological outcomes with a 5-year OS of 88.9%, a 5-year DSS of 95.5% and a 5-year PFS of 90.4%, and are confirming the reported positive results for HPV-OPSCC [12, 25–27].

Previous studies argued that HPV-HNCUP and HPV-OPSCC are the same disease, as both showed similar risk profiles and survival data [15, 28]. In contrast, a comparison of the survival data of the two diseases in our cohort shows a clear difference with a worse survival for HPV-HNCUP patients.

A brief look at our patients’ history of smoking revealed that 18 out of 21 patients (85.7%) with HPV-HNCUP and 68 out of 84 patients (81%) with HPV-OPSCC were smokers. The data from Tribius et al. showed that smoking is a negative prognostic factor for patients with HPV-positive cancers [29] and hence an explanation for the worse results of the HPV-HNCUP patients. However, this is contradicted by the fact that there are almost equal numbers of smokers in both groups. This means that the risk is equally distributed in both cohorts and thus supports the theory that the diseases mentioned are not the same disease.

Ross et al. showed comparable survival data for early-stage HPV-OPSCC and HPV-HNCUP and proposes to include both patients in clinical trials investigating the role of treatment deintensification [28]. In our cohort, 95.2% of HPV-OPSCC patients had a UICC stage I/II and yet survival was significantly worse for HPV-HNCUP patients, all of whom had a UICC stage I/II. The favorable prognosis of HPV-OPSCC in comparison to HPV negative OPSCC has already led to discussion on de-escalation of therapy in the form of clinical studies [30–32]. However, it is controversial whether these results can be readily applied to every patient and especially to patients with HNCUP.

A limitation of our study is the retrospective study design. Nevertheless, it should be emphasized that this is a homogeneous patient population treated at a single tertiary referral cancer center. Furthermore, all patients received the same combined therapy consisting of surgical and adjuvant therapy. Accordingly, the results cannot be transferred to patients who received a single therapy modality, i.e., surgery or radiation alone, as this was beyond the scope of this study.

Another limitation is the fact that the HPV status was determined only by the p16-status and not additionally by HPV-DNA. A routine determination of HPV-DNA from the specimens is not done in our clinic and subsequently there is a risk that a determination of p16INK4a alone is not sufficient to diagnose HPV-associated HNCUP, with the consequence that a possible primary is not located in the oropharynx and the corresponding tumor stage of the patients would be higher. Other risks for bias are the increased number of extranodal extension in the HPV-HNCUP cohort, the associated more radical approach to neck dissection, and the overall lower number of lymph nodes harvested. It must be taken into account that the HNCUP patients only received a median removal of 16 lymph nodes and the definition of an adequate neck dissection includes 18 lymph nodes [33], a number which originates from head and neck cancer of known origin and cannot be easily transferred to HNCUP. Also, significantly more lymph nodes were removed in the HPV-OPSCC patients, since most patients received a neck dissection bilaterally. Furthermore other risk factors such as perineural and lymphovascular invasion were not analyzed, as these were not available for HNCUP patients. All of these factors must be taken into account when interpreting the results.

Since HPV-HNCUP cases are rare, prospective studies are difficult. This is also reflected in the literature. Furthermore, the existing studies often show inhomogeneous patient populations with different treatment modalities. Nevertheless, this is an important topic that should be further investigated. One way to increase the number of patients and thus the knowledge about this disease in the long term is to conduct multicenter, prospective studies.

## Conclusion

The results of this study demonstrate distinct superior oncologic outcomes of patients with a HPV-associated oropharyngeal cancer compared to matched HPV-HNCUP patients determined by propensity score matching. Accordingly, our data does not support the hypothesis of both cancers representing the same disease. Prospective multicenter studies are imperative to investigate the nature of this rare disease of carcinoma of unknown origin associated with HPV.

### Supplementary Information

Below is the link to the electronic supplementary material.

Supplementary file1 (DOCX 13 KB)

## Data Availability

The datasets used and/or analyzed during the current study are available from the corresponding author on reasonable request.

## References

[1] Chaturvedi AK (2011). Human papillomavirus and rising oropharyngeal cancer incidence in the United States. J Clin Oncol.

[2] Chaturvedi AK (2008). Incidence trends for human papillomavirus-related and -unrelated oral squamous cell carcinomas in the United States. J Clin Oncol.

[3] Shield KD (2017). The global incidence of lip, oral cavity, and pharyngeal cancers by subsite in 2012. CA Cancer J Clin.

[4] Marur S (2010). HPV-associated head and neck cancer: a virus-related cancer epidemic. Lancet Oncol.

[5] Ang KK (2010). Human papillomavirus and survival of patients with oropharyngeal cancer. N Engl J Med.

[6] Motz K (2016). Changes in unknown primary squamous cell carcinoma of the head and neck at initial presentation in the era of human papillomavirus. JAMA Otolaryngol-Head Neck Surg.

[7] Jensen DH (2014). Human papillomavirus in head and neck squamous cell carcinoma of unknown primary is a common event and a strong predictor of survival. PLoS ONE.

[8] Keller LM (2014). p16 status, pathologic and clinical characteristics, biomolecular signature, and long-term outcomes in head and neck squamous cell carcinomas of unknown primary. Head Neck.

[9] Kobayashi K (2014). Clinical features of human papilloma virus-related head and neck squamous cell carcinoma of an unknown primary site. ORL J Otorhinolaryngol Relat Spec.

[10] Axelsson L (2017). Prognostic factors for head and neck cancer of unknown primary including the impact of human papilloma virus infection. J Otolaryngol Head Neck Surg.

[11] Gillison ML (2015). Epidemiology of human papillomavirus-positive head and neck squamous cell carcinoma. J Clin Oncol.

[12] Wang MB (2015). HPV-positive oropharyngeal carcinoma: a systematic review of treatment and prognosis. Otolaryngol Head Neck Surg.

[13] Winter SC (2017). Trans-oral robotic assisted tongue base mucosectomy for investigation of cancer of unknown primary in the head and neck region. The UK experience. Clin Otolaryngol.

[14] Golusinski P (2019). Evidence for the approach to the diagnostic evaluation of squamous cell carcinoma occult primary tumors of the head and neck. Oral Oncol.

[15] Schroeder L (2020). HPV driven squamous cell head and neck cancer of unknown primary is likely to be HPV driven squamous cell oropharyngeal cancer. Oral Oncol.

[16] Maghami E (2020). Diagnosis and management of squamous cell carcinoma of unknown primary in the head and neck: ASCO guideline. J Clin Oncol.

[17] Robbins KT (1991). Standardizing neck dissection terminology. Official report of the Academy's Committee for Head and Neck Surgery and Oncology. Arch Otolaryngol Head Neck Surg.

[18] Gil Z (2009). Lymph node density is a significant predictor of outcome in patients with oral cancer. Cancer.

[19] Brierley JD, GM, Wittekind C (2016) TNM classification of malignant tumors. 8th ed. Wiley‐Blackwell, Hoboken

[20] Ho DE, Imai K, King G, Stuart EA (2011). MatchIt: nonparametric preprocessing for parametric causal inference. J Stat Softw.

[21] Field A (2005). Discovering statistics using SPSS.

[22] Schroeder L (2017). Human papillomavirus as prognostic marker with rising prevalence in neck squamous cell carcinoma of unknown primary: a retrospective multicentre study. Eur J Cancer.

[23] Schroeder L (2018). Antibodies against human papillomaviruses as diagnostic and prognostic biomarker in patients with neck squamous cell carcinoma from unknown primary tumor. Int J Cancer.

[24] Ren J (2019). Human papillomavirus and p16 immunostaining, prevalence and prognosis of squamous carcinoma of unknown primary in the head and neck region. Int J Cancer.

[25] Dayyani F (2010). Meta-analysis of the impact of human papillomavirus (HPV) on cancer risk and overall survival in head and neck squamous cell carcinomas (HNSCC). Head Neck Oncol.

[26] O'Rorke MA (2012). Human papillomavirus related head and neck cancer survival: a systematic review and meta-analysis. Oral Oncol.

[27] Saulle R (2015). Human papillomavirus and cancerous diseases of the head and neck: a systematic review and meta-analysis. Oral Dis.

[28] Ross RB (2018). A matched comparison of human papillomavirus-induced squamous cancer of unknown primary with early oropharynx cancer. Laryngoscope.

[29] Tribius S (2012). HPV status in patients with head and neck of carcinoma of unknown primary site: HPV, tobacco smoking, and outcome. Oral Oncol.

[30] Dalianis T (2014). Human papillomavirus and oropharyngeal cancer, the epidemics, and significance of additional clinical biomarkers for prediction of response to therapy. Int J Oncol.

[31] Mirghani H (2015). Treatment de-escalation in HPV-positive oropharyngeal carcinoma: ongoing trials, critical issues and perspectives. Int J Cancer.

[32] Sivars L (2016). Human papillomavirus as a diagnostic and prognostic tool in cancer of unknown primary in the head and neck region. Anticancer Res.

[33] Divi V (2016). Establishing quality indicators for neck dissection: correlating the number of lymph nodes with oncologic outcomes (NRG Oncology RTOG 9501 and RTOG 0234). Cancer.

