# Supplementary material for: A comparison between p16-positive head and neck cancer of unknown primary (HPV-HNCUP) and oropharyngeal squamous cell carcinoma (HPV-OPSCC): are they the same disease?
Source: Eur Arch Otorhinolaryngol. 2023 Jul 28;280(12):5489–97. doi: 10.1007/s00405-023-08115-5 (PMC10620243; doi:10.1007/s00405-023-08115-5)
Supplement: Supplementary file 1 — Supplementary file1 (DOCX 13 KB) [file 405_2023_8115_MOESM1_ESM.docx]

|  | **Pre PSM** | | | **Post PSM** | | |
| --- | --- | --- | --- | --- | --- | --- |
|  | CUP  (n= 21) | OPSCC  (n= 126) | Statistical comparison | CUP  (n= 21) | OPSCC  (n= 84) | Statistical comparison |
| Gender (n, %)  Male  Female | 17 (81.0%)  4 (19.0%) | 91 (72.2%)  35 (27.8%) | p = 0.402, phi = 0.069 | 17 (81.0%)  4 (19.0%) | 64 (76.2%)  20 (23.8%) | p = 0.642, phi = 0.045 |
| Age (mean years ± SD) | 59.48 ± 9.90 | 59.98 ± 9.33 | p = 0.831, d = 0.053 | 59.48 ± 9.90 | 58.54 ± 8.66 | p = 0.693, d = 0.105 |
| Nodal stage (n, %)  pN0  pN1  pN2  no ND | 0 (0%)  18 (85.7%)  3 (14.3%)  0 (0%) | 15 (11.9%)  90 (71.4%)  16 (12.7%)  5 (4%) | p = 0.276, Cramer’s V = 0.162 | 0 (0%)  18 (87.7%)  3 (14.3%)  0 (0%) | 2 (2.4%)  68 (81.0%)  14 (16.7%)  0 (0%) | p = 0.739, Cramer’s V = 0.076 |
| Adjuvant treatment modality (n, %)  Radiation therapy  Chemoradiation therapy | 4 (19.0%)  17 (81.0%) | 41 (32.5%)  85 (67.5%) | p = 0.214, phi = 0.102 | 4 (19.0%)  17 (81.0%) | 20 (23.8%)  64 (76.2%) | p = 0.642, phi = 0.045 |
| Noxious agents  Smoking | 18/21 (85.7%) | 78/119 (65.6%) | **p = 0.066, phi = 0.155** | 18 (85.7%) | 68 (81.0%) | p = 0.612 phi = 0.049 |

Tab. S1: Patients´ characteristics before and after Propensity Score Matching regarding matching variables.

Abbreviations: PSM = Propensity Score Matching; SD = standard deviation; UICC = International Union Against Cancer; ASA = American Society of Anesthesiologists.
